# Preparation, Physicochemical Characterization and *In-vitro* Dissolution Studies of Diosmin-cyclodextrin Inclusion Complexes

**Published:** 2014

**Authors:** Fengwei Ai, Yingli Ma, Jiayu Wang, Yanfeng Li

**Affiliations:** a *School of Pharmacy, Xuzhou Medical College, Xuzhou 221004, China. *; b *School of Pharmacy, Heilongjiang University of Chinese Medicine, Haerbin 150040, China.*

**Keywords:** Diosmin, Cyclodextrins, Solubility, Inclusion complex, Dissolution rate

## Abstract

Diosmin, a vascular-protecting agent, is practically insoluble in water, and its oral absorption is limited by its extremely low dissolution rate. In this study, β-cyclodextrin (βCD) and 2-hydroxypropyl-β-cyclodextrin (HPβCD) were obtained to improve the solubility and dissolution rate of diosmin. Phase solubility studies of diosmin with βCD and HPβCD in distilled water were conducted to characterize the complexes in liquid state. The solid-state characterization of the complexes prepared with different methods was performed by fourier transform-infra red spectroscopy (FTIR), optical microscopy analyses, and differential scanning calorimetry (DSC). Dissolution studies were carried out in distilled water using US pharmacopeia dissolution rate testing equipment. The complexation of diosmin with βCD and HPβCD both indicated an A_L_ type of phase-solubility diagrams, and the apparent stability constants (Kc) was found to be 222.13 and 200.08 M^−1^, respectively. The Kc values indicated the βCD and HPβCD showed the similar equal complexation ability with diosmin, HPβCD provided higher solubility for diosmin due to its higher water solubility. The dissolution studies suggest that the inclusion complexes provide higher dissolution rate compared with the physical mixtures and the drug alone. Furthermore, the inclusion complex prepared by freeze drying method presented higher dissolution rate than kneading method.

## Introduction

In the drug discovery field, there are a seemingly infinite number of potential drug candidates to be synthesized and screened using combinatorial chemistry and systematic high throughput screening technology, respectively (1). However, about 60% of molecules obtained directly from synthesis are usually poorly soluble in water (2). Poorly water-soluble drugs often show low bioavailability when administered orally, since the absorption of the drugs in the gastrointestinal tract can usually be a rate-limiting step (3, 4). Thus, many molecules that are biologically active *in-vitro* are ineffective *in-vivo* as a result of their limited solubility and slow rate of dissolution (5, 6).

During the past few decades there has been wide interest in exploring new techniques for increasing drug solubility. The inclusion complex with cyclodextrin (CyD) is one of the most widely used approaches for improving dissolution rate of the drugs (7, 8). The natural CyDs are cyclic oligosaccharides made up of 6–8 dextrose units (α-, β-, γ-cyclodextrins, respectively) joined through 1-4 bonds. The researchers also prepared numerous CyD derivatives by substituting the hydroxy groups of natural CyDs with other functions. The inner cavity of these molecules is relatively lipophilic and the outer surface relatively hydrophilic. Cyclodextrins are able to incorporate apolar molecules or parts of molecules inside their hydrophobic cavity (9). Of the native and modiﬁed CyDs, HPβCD is most widely used in the pharmaceutical industry, it is readily available, has high solubility (> 50%) in water, and moreover, it provides good inclusion complexation with a wide range of drugs and maximal *in-vivo* safety for various biomedical uses (10-12).

**Figure 1:**
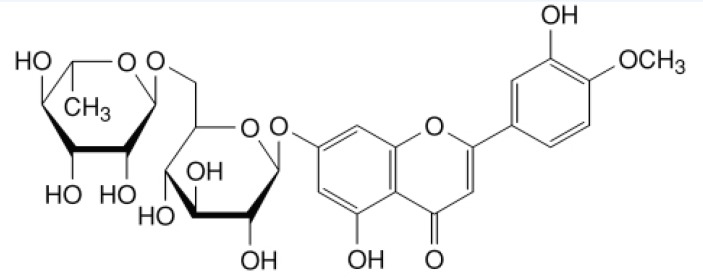
The structure of diosmin

Diosmin(7-[[6-O-(6-Deoxy-α-L-mannopyranosyl)-β-D-glucopyranosyl]oxy]-5-Hydroxy-2-(3-hydroxy-4-methoxyphenyl)-4H-1-ben-zopyran-4-one, Figure 1), is a natural flavonoid glycoside that can be isolated from various plant sources or derived from the flavonoid hesperidin (13). It is considered to be a vascular-protecting agent has been used for the treatment of chronic venous insufficiency (CVI) (14), haemorrhoids (15), lymphedema (16), and venous leg ulcers (17). As a flavonoid, diosmin also exhibits anti-inflammatory (18), free-radical scavenging (19), radioprotective effect (20), and antimutagenic (21) properties. However, diosmin is practically insoluble in water, and currently marketed as tablets containing the drug in crystalline form with controlled particle size. Therefore, it is necessary to design a formulation possible to increase the dissolution behavior of diosmin in water to improve the oral absorption and bioavailability. Thus, we decided to use CyDs as pharmaceutical excipients for this purpose. To our knowledge, there has been no report examining the interaction between diosmin and βCD/HPβCD, nor on the enhancing effects of HPβCD/βCD on the solubility of diosmin. In present study, the influence of βCD/HPβCD concentration on the solubility of diosmin was evaluated by phase solubility studies. HPβCD was obtained to prepare inclusion complexes due to its better water solubility. The diosmin-HPβCD inclusion complexes were prepared by freeze drying and kneading method (22, 23). The physical characteristics of the inclusion complexes, physical mixtures and diosmin alone were studied using FTIR, Optical Microscopy and DSC. In addition, the dissolution profiles of diosmin-HPβCD inclusion complexes were investigated and compared with those of their physical mixtures and diosmin alone. 

## Experimental

*Materials*

Diosmin was obtained from Shanxi Huike Botanical Development Co., *Ltd*. (Xian, China). βCD and HPβCD were obtained from Shandong Xinda Fine Chemical Co., *Ltd*. (Xincheng, China), and the average degree of substitution of 2-hydroxypropyl groups in HPβCD was determined to be 1.19. Methanol was HPLC grade, other chemicals and solvents were of analytical reagent grade and double-distilled water was used throughout the study.

*Assay for* *Diosmin*

The assay for diosmin in this study was developed and validated based on previous reports (24) by high performance liquid chromatography (HPLC) on an Agilent 1260 HPLC system. Separations were performed on a Zorbax Eclipse XDB C18 150×4.6-mm column (Agilent Technology, USA) at room temperature. The mobile phase consisted of water/methanol in the ratio of 50:50 (v/v) with an isocratic flow rate of 1.0 mL/min. The detector was set at 275 nm and the sample injection volume was 10 μL. The chromatographic method was established to be accurate, precise and selective for the analysis of diosmin.

*Phase solubility studies*

Phase solubility studies in distilled water were conducted according to the method of Higuchi and Connors (25). Excess amounts of diosmin were added to a series of glass vials containing 10.0 mL of distilled water. Increasing amounts of various CyDs (βCD: 0.2%, 0.4%, 0.6%, 0.8%, 1.0%; HPβCD: 1%, 2.5%, 5%, 7.5%, 10%, 15%, 20% w/v) were then placed in each sample. These suspensions were shaken at 25 °C in a reciprocating water bath. After equilibrium was reached (about 96 hours), an aliquot was centrifuged, and then the supernatant was ﬁltered through a 0.45-µm PES disc filter (MEMBRANA, German), after appropriately diluted, the concentration of diosmin was analyzed by HPLC. Solubility diagrams were constructed by plotting the molar concentration of diosmin dissolved (solubility) versus the molar concentration of the cyclodextrins. The apparent stability constant (Kc) of the complexes was calculated from the slope of the phase-solubility diagrams using the following equation (25):

Kc= slope/S_0_ (1–slope)                    Equation (1)

The slope is obtained from the initial straight-line portion of the plot of diosmin concentration against cyclodextrins concentration.

S_0_ is intercept of the linear portion of the phase solubility diagram.

*Preparation of solid Inclusion complexes*

Various diosmin-HPβCD formulations were prepared in a 1:1 molar ratio based on the results of the preliminary phase solubility studies.

(a). Physical mixture (PM): physical mixture was prepared by homogeneous blending of previously sieved and weighed diosmin and HPβCD in a mortar in 1:1 molar ratio.

(b). Kneading Method(KN): The solid disomin complexes with cyclodextrins in a molar ratio of 1:1 was prepared by adding small amount of water to HPβCD placed in a mortar and mixing thoroughly to obtain a homogeneous paste. Then, the sieved diosmin powder was slowly added and the mixture was kneaded for about 60 min. The resulting paste was dried in an oven at 40 °C. The dried complex was pulverized and sieved through a 100 mesh sieve. It was then stored in a desiccator for use.

(c). Freeze Drying Method (FD): freeze–dried (FD) product was prepared by dissolving the diosmin in small amount of DMSO and adding the stoichiometric amount of an aqueous solution of HPβCD. The resulting solutions were frozen at −70 °C (Boyikang DW-400, China) and lyophilized in a freeze dryer (Boyikang FD-1, China). The lyophilized powder was passed through a 100-mesh sieve and stored in a desiccator for use.

*Fourier Transform-Infra Red Spectroscopy (* *FT* *IR)*

Infrared spectra of diosmin, β-CD and their binary products were obtained with Shimadzu FTIR-8400s spectrophotometer. Complex formation was evaluated by comparing the IR spectra of the solid complexes and of the physical mixture containing the same amount of diosmin. The inclusion complexes and physical mixtures contain the same amount of diosmin, the ratio of diosmin and KBr was constant in the experiment in order to evaluate the IR spectra. Accurately weighed of various samples and KBr were previously ground and mixed thoroughly, compressed and analysed on a spectrophotometer. The scanning range was 4000–400 cm^−1^ and the resolution was 2 cm^−1^.

*Optical microscopy*

The surface morphology of diosmin, HPβCD and their binary systems were analyzed by using an optical microscope (OLYMPUS-CX21FS1). The samples were mounted on a glass slide, viewed under normal light (26) and pictures were taken with a GY-5000 camera (Nanjing guangyou optical & electrical instrument CO.LTD).

*Differential* *S* *canning* *C* *alorimetry* *(DSC)*

The thermal analysis of pure diosmin, β-CD and their binary products were carried out using a Shimadzu DSC-60 instrument. Various samples (5 mg) were sealed in aluminum pans and the DSC thermograms were recorded at a heating rate of 10 °C/min from 30 °C to 320 °C. An empty pan served as reference and indium was used to calibrate the temperature.

*In-vitro dissolution studies*

The dissolution behaviors of the diosmin-HPβCD complexes were compared with those of pure diosmin and physical mixture. The dissolution rate studies were performed using a dissolution rate test apparatus (ZRS-8, Radio Factory of Tianjin University, China) which used a paddle method. The samples, corresponding to 10 mg of diosmin were analysed in 900 mL water. The stirring speed was 50 rpm, and the temperature was maintained at 37 ± 0.5 °C. At fixed time intervals (2, 6, 10, 15, 20, 30, 40, 60, 90, and 120 minutes), the samples (10 mL) were withdrawn with a syringe, filtered using a 0.45-μm PES disc filter. After appropriately diluted, the concentration of diosmin was analyzed by HPLC as described above. Meanwhile, 10 mL of fresh release media was added in order to maintain the volume of dissolution medium. Dissolution experiments were carried out in triplicate. The dissolution profiles were evaluated on the basis of the dissolution efficiency parameter at 10 min (DE_10_, %), 30 min (DE_30_, %) and 120 min (DE_120_, %). The dissolution efficiency parameters were calculated from the area under the dissolution curves and expressed as a percent of the area of the rectangle described by 100% dissolution in the same time period (27).

## Results and Discussion

*Phase* *solubility studies*

**Figure 2:**
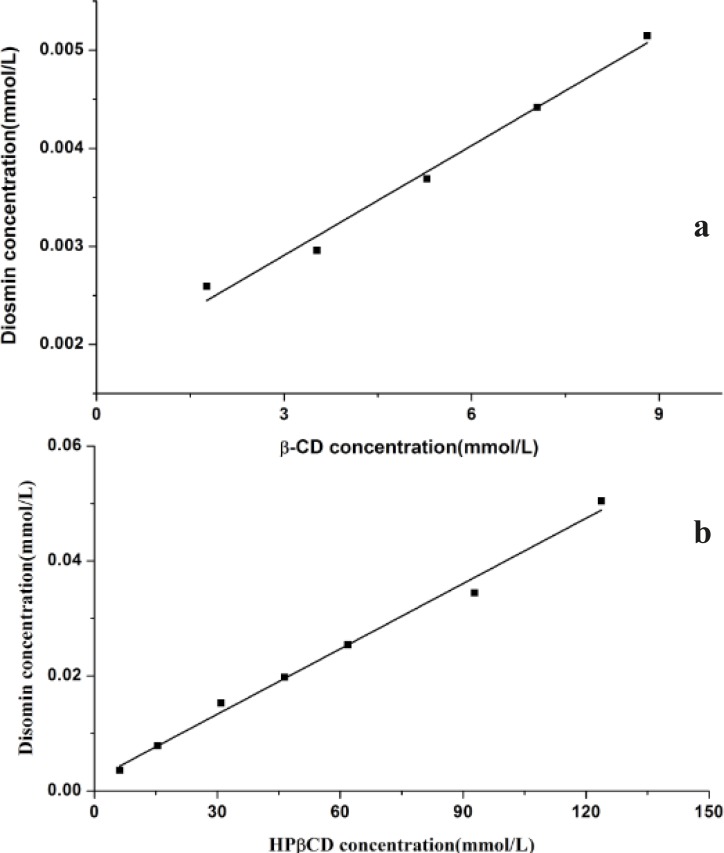
Phase solubility diagrams of diosmin-βCD(a) and diosmin-HPβCD(b), at 25 °C in water.

According to Higuchi and Connors, phase solubility studies can provide not only the stability constant of the complex but also to give insight into the stoichiometry of the complex at equilibrium. The phase solubility diagrams at 25 °C were obtained by plotting the apparent equilibrium concentrations of the drug against βCD or HPβCD concentrations and were shown in Figure 2. The Kc values of the inclusion complexes derived from the phase diagram of the study were 222.13 and 200.08 M^−1^ for βCD and HPβCD, respectively.

The increase in solubility of diosmin occurred as a linear function of βCD/HPβCD concentration over the entire concentration range studied. The linear relation between diosmin solubility and βCD/HPβCD concentration indicates an A_L_-type phase-diagram and characteristic of 1:1 complexation, deﬁned by Higuchi and Connors. The Kc values indicate that both βCD and HPβCD forming a stable inclusion complex with diosmin in water. Moreover, the βCD and HPβCD show nearly equal complexation ability with diosmin by comparing their Kc values.

*Infrared spectroscopy*

**Figure 3:**
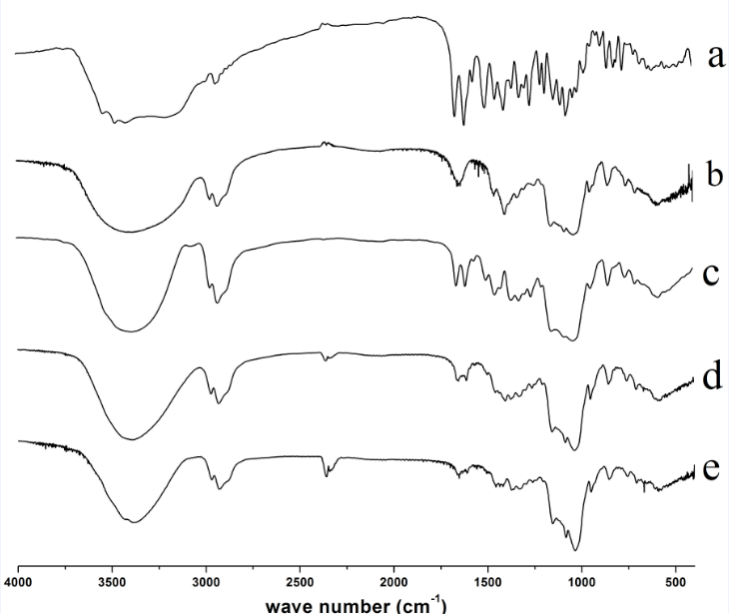
IR spectra of Diosmin (a), HPβCD (b), physical mixture (PM) (c), kneaded (KN) (d) and freeze–dried (FD) products (e).

IR spectra of diosmin, HPβCD, physical mixture and diosmin-HPβCD solid complexes formed by kneading, and freeze drying techniques were presented in Figure 3. The IR spectrum of diosmin is shown in Figure 3a. Diosmin shows a characteristic carbonyl absorption band at 1661.7 cm^−1^, assigned to aromatic ketonic carbonyl stretching (C=O vibration), that at 1610.6 cm^−1^, 1500.7 cm^−1^ to the stretching of the C=C bond in the aromatic ring. HPβCD spectrum (Figure 3b) shows characteristic absorption bands at 1644.3, 1401 cm^−1^. In addition, a broad absorption band around 3380.3 cm^-1 ^due to OH stretching, a large region which displays distinct peaks in the region of 900-1200 cm^−1^. The physical mixture exhibits spectrum (Figure 3c) corresponding to a superposition of their parent components (Figure 3a and b, respectively) with no signiﬁcant shift in the major peaks, indicating that no interaction had occurred as a result of simply physically mixing the free drug with HPβCD. However, for the kneaded product (Figure 3d) the drug characteristics peaks were smaller. The freeze-dried product (Figure 3e) spectra also showed differences, with absence of the characteristic absorption bands of diosmin. Changes in the characteristic bands of pure drug confirm the existence of the complex as a new compound with different spectroscopic bands.

*Optical microscopy*

**Figure 4:**
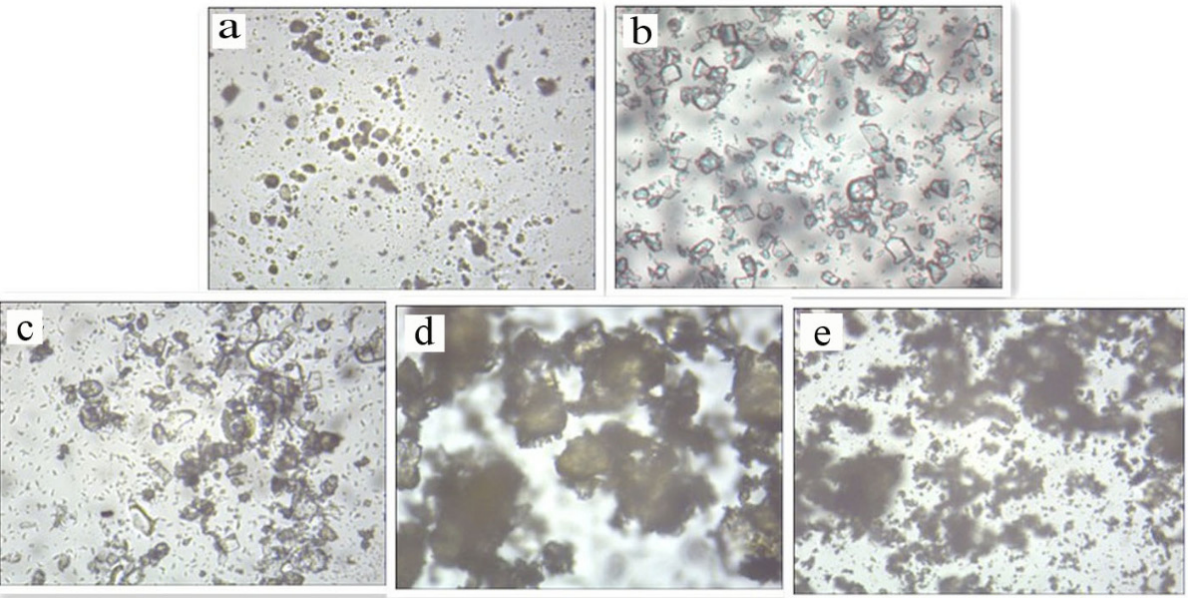
Optical microscopy of diosmin (a), HPβCD (b), physical mixture (c), kneaded (d) and freeze-dried (e) products at magniﬁcation of ×100.

A morphological analysis of diosmin, HPβCD, physical mixture and diosmin-HPβCD solid complexes were performed using Optical microscopy. The photomicrographs of samples are shown in Figure 4. The image of diosmin (Figure 4a) shows these powders are yellow-green and amorphous, the image of HPβCD (Figure 4b) indicates that these powders are flake and crystalline, these characteristic patterns for diosmin and HPβCD crystals are presented in the physical mixture (Figure 4c), where both diosmin and HPβCD crystals can be detected, and the size of HPβCD crystals is larger than diosmin. Whereas the diosmin-HPβCD inclusion complex shows a completely different arrangement: an amorphous powder that difference with the diosmin or HPβCD crystal patterns. Both the kneaded product (Figure 4d) and the freeze-dried products (Figure 4e) showed an amorphous powder, but the particles of the kneaded product are larger.

*Differential scanning calorimetry*

**Figure 5:**
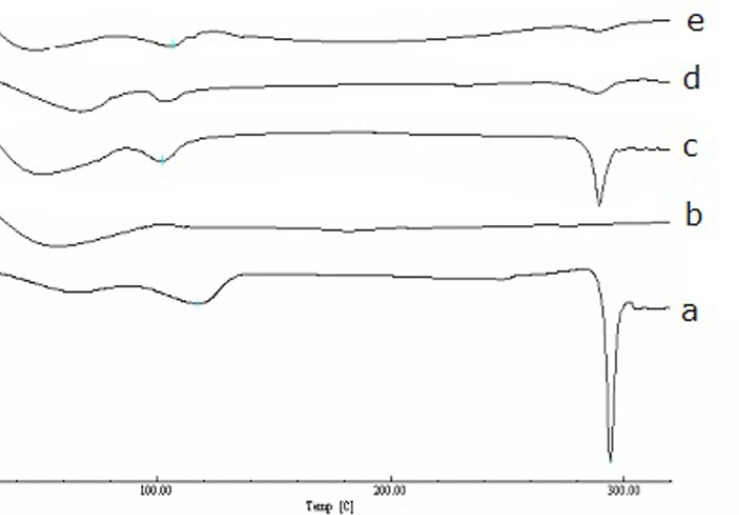
The DSC thermograms of diosmin (a), HPβCD (b), physical mixture (c), KN (d) and FD products (e).

The DSC thermograms of diosmin, HPβCD, and the binary systems of diosmin with HPβCD are shown in Figure 5. The DSC thermogram of diosmin exhibits a sharp endothermic peak at 293.05 °C indicating the melting point and a broad endothermic peak at 116.54 °C corresponding to loss of water. The DSC curve of HPβCD exhibits a very broad endothermal phenomenon between 40 °C and 90 °C due to the release of water molecules. For the physical mixture of diosmin and HPβCD, the endothermic peak at 293.05 °C and broad peaks between 40 °C and 120 °C corresponding to diosmin and HPβCD peaks. For the kneaded drug-HPβCD inclusion complex, the DSC thermograms have a much smaller endothermic peak than the physical mixture corresponding to the melting point of diosmin. However, in the freeze-dried mixture, the endothermic peak was absent. These changes in DSC thermograms showed an interaction of diosmin with HPβCD in the inclusion complexes.

*Dissolution studies*

**Figure 6:**
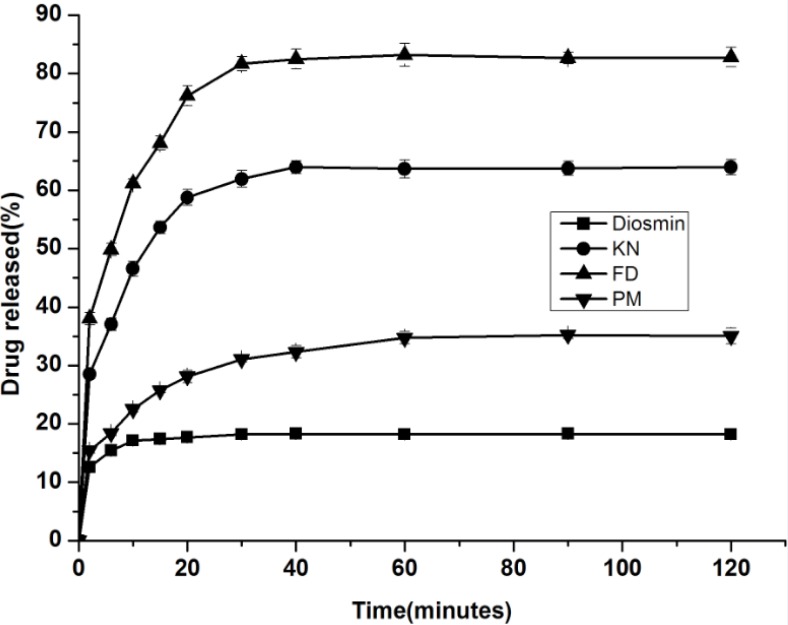
Dissolution curves of complexes, drug alone and physical mixture in water

Dissolution profiles for the drug, physical mixture, and inclusion complex (FD and KN) were presented to demonstrate the influence of a CyD on the drug dissolution. The dissolution rate profiles of diosmin, inclusion complexes and the physical mixture are shown in Figure 6. The solubility profiles of the complex showed that 82.8% (FD product) and 64.0% (KN product) drug was released in 2 hours compared to 18.2% that of the pure diosmin. For further evaluation, three dissolution efficiency parameters (DE_10_, DE_30 _and DE_120_) were measured for all products studied (Table 1). 

The Figure 6 illustrates that the solubility of complexes is higher compared with the drug alone, and the release of the inclusion complex was strongly affected by the method of formulation. The FD products exhibited the best dissolution properties and were followed by the kneaded product and physical mixtures, respectively. This result is attributed to the FD product providing smaller particle size. The dissolution efficiency values also support the above result. The DE_120 _of physical mixture was about 2-folds higher than the drug alone, and the DE_10_, DE_30_ and DE_120 _of inclusion complex were about 3, 4-folds higher than the drug alone. 

**Table 1:** The dissolution efficiency of diosmin, physical mixture (PM), kneaded (KN) and freeze-dried (FD) Products.

**Products**	**DE**_10_ ** (%)**	**DE**_30_ ** (%)**	**DE**_120_ ** (%)**
**Diosmin**	13.37 ± 3.51	16.25 ± 4.15	17.76 ± 3.43
**Physical mixture(PM)**	16.50 ± 2.31	23.87 ± 1.48*	31.73 ± 3.87**
**Kneaded(KN)**	32.68 ± 2.81**	48.75 ± 2.29***	59.97 ± 2.05***
**Freeze-Dried(FD)**	43.58 ± 1.24***	63.63 ± 1.47***	77.97 ± 2.000***

Values are mean ± SD.

* *p*<0.05,

** *p*<0.01,

*** *p*<0.001 compared with pure diosmin.

## Conclusions

The inclusion complexes in liquid state of diosmin with βCD and HPβCD were characterized with phase solubility studies, the phase solubility diagram of diosmin with βCD and HPβCD both showed A_L_-type diagrams with characteristic of 1:1 complexation, and Kc values were found to be 222.13 M^−1^ for βCD and 200.08 M^−1^ for HPβCD. Meanwhile, the solubility of diosmin could be increased by 286.05% at 8.81 mmol/L of βCD and by 2521.52% at 123.73 mmol/L of HPβCD, respectively. The solid inclusion complex of diosmin with HPβCD was obtained by kneading and freeze drying, the DSC, FTIR and optical microscopy analyses suggested that diosmin could form stable inclusion complex in a 1:1 molar ratio. The dissolution studies suggested that the inclusion complexes of diosmin with HPβCD provide higher dissolution rate comparing with the physical mixtures or the drug alone, additionally, the inclusion complex prepared by freeze drying method shows higher dissolution rate than by kneading method.
